# The Dangers of Being Old in Rural Tanzania: A Call for Interventions for Strengthening Palliative Care in Low-Income Communities

**DOI:** 10.3389/fragi.2022.888396

**Published:** 2022-04-25

**Authors:** Kahabi Ganka Isangula

**Affiliations:** ^1^ School of Nursing and Midwifery, The Aga Khan University, Dar Es Salaam, Tanzania; ^2^ Inspire Medical Services Dispensary, Shinyanga, Tanzania

**Keywords:** palliative care, elderly, old age, rural, Shinyanga, Tanzania, Africa, low-income countries

## Introduction

Tanzania is currently implementing political reforms that, with time, are expected to improve quality of life among individuals regardless of their age. This improvement in quality of life, consequently prolonged life expectancy implies that the aging population will significantly increase in a few years to come. Research projects that the number of people aged 60 years and above will increase from the current 4-percent to 10-percent of the total population by 2050 (United Republic of Tanzania, 2003; Kivelia and Kirway, 2011; Morrissette and Wane, 2012). The projected increase suggests that the older population will be at a disadvantage because majority of older people in Tanzania resides in rural areas where financial support and access to social services including Primary Health Care (PHC) services is often limited (Kivelia and Kirway, 2011; Morrissette and Wane, 2012; Sanga, 2013; Musa, 2016).

The older population in Tanzania continues to form a significant percentage of people seeking care for chronic diseases in PHC facilities. Reports indicate that older patients (aged 60 years and above) contribute significantly to aging related diseases, for instance, cardiovascular diseases (United Republic of Tanzania, 2007; UNFPA, 2009; Isangula and Meda, 2017). The prevalence of hypertension in Tanzania for example, is much higher among older people as compared to much younger population (Isangula and Meda, 2017). This calls for strong policies that seek to ensure that the older population are well cared for within rural PHC facilities in their final years of life.

While aged care institutions are the cornerstone of Western care, similar institutions are lacking in low-income rural African communities. Consequently, older people in rural settings, mostly uneducated, uninsured, unable to work and often charged with the care of grandchildren, continue to rely on sons and daughters (if any) and neighbors to meet their daily socioeconomic needs (Sanga, 2013; Musa, 2016; United Republic of Tanzania, 2007; UNFPA, 2009). This dependency often contributes to persistent limited access to quality nutrition and medical care among the older population when family members lack the financial capacity to support them. This brings us the question of how better we can, as a country, care for our aging population as part of ensuring they enjoy their final years of life. This paper examines the challenges faced by the older population in a low-income rural community of Shinyanga in relation to access to the much-needed palliative care.

## Materials and Methods

This paper is descriptive in nature, structured around the personal experiences of caring for patients with old age in a rural community to highlight on the sufferings they continue to face and the demand for palliative care.

### Settings

The opinions are based on experiences that occurred in Shinyanga region. Isangula (2018) offers a detailed description of this setting. Briefly, Shinyanga region extends about 160 km from the southern part of Lake Victoria’s shoreline, forming part of what used to be known as the Sukuma land. The region is administratively divided into five districts: Shinyanga MC, Shinyanga DC, Kishapu DC, Kahama MC and Kahama DC. The 2012 national census documented the region’s population of 1,534,808 (or 81 people per square kilometer), with >95-percent rural occupancy and about 5-percent of the older population.

PHC services have been reported as inadequate in Shinyanga (Isangula, 2018). People rely on predominantly few public owned PHC facilities and traditional healers who are considered very active compared to other regions. With 161,391 people in Shinyanga Council, for instance, only 28 PHC facilities are available, most of which are located in the urban part (covering about 5% of the population) leaving the majority with minimal to no access to quality health care services in rural areas.

## Results

A PHC facility was established in early October in 2018 for the purpose of ensuring access to affordable health care services among low income rural population in Shinyanga. Since then, the author, a medical doctor by profession who has just completed PhD studies in Australia, started offering medical care including home based palliative care. There have been a lot of interesting medical cases, for instance, Mondor’s disease arising from intense-hoe farming (Isangula, 2019) which reflects the prevailing microeconomic activities in this rural community. Chronic diseases such cancer, hypertension and diabetes that have a negative impact on the older population are also common (Isangula, 2018). For instance, of 120 people who attended a free health assessment week as part of the official opening of the PHC facility in October 2018, 39-percent were previously undiagnosed hypertensive, of which, 90-percent were of age 60 years and above. Since October 2018, the author had personally offered care to more than 630 clients with chronic diseases aged more than 60 years up to October 2021. Below, forming part of this opinion paper, two cases are described as exemplars.

The first case identified with the need of the continued palliative care was patient Limi (*not the real name*), female, 68 years of age presented in December 2020 with a history of frequent nausea and vomiting, loss of appetite, fatigue and weakness, sleep problems, changes in urination patterns, swelling of feet and ankles, persistent itching, difficulty in breathing and hypertension. These features have been associated with an end-stage renal disease (Somma et al., 2013). We could not treat the renal disease as we did not have the resources to offer renal replacement therapy which is often recommended in similar cases (Somma et al., 2013). The complications associated with the disease itself and the old age included inability to move, therefore always bedridden that lead to painful and occasionally infected pressure sores with deep tissue and bone involvement that further raised the suspicion of Osteomyelitis and required antibiotic treatment and daily dressing. Pressure sores kept re-appearing on one side as we concentrated on management of the infected sores on the other side. We did not have the resources and neither did the family have the financial capacity to meet the cost of histopathological examination as well as tissue and bone biopsy. Histopathological examination and bone biopsy are considered the gold standard for detection of osteomyelitis in some studies (Yoshikawa et al., 2002). Some of health care providers within my team (who had come from public facilities) did not have basic palliative care skills, therefore, we had to conduct a brief training using materials accessed from African Palliative care organization’s website (https://www.africanpalliativecare.org). We then continued to offer homebased care that was frequently required. However, given that the family had to meet some of the cost, home based care later became too expensive for them even when the visitation fee was waived. Access to pain medication was also limited due to restrictive policies in the country (Nanney et al., 2010), therefore, conventional medications were frequently used. After few months of living in pain and relying on our team’s support, Limi died. This raises the question as to how many other patients like Limi continues to die in this rural community because they cannot access palliative care?

A second case is a 73-year-old male, a former smoker and alcoholic presenting in August 2021 with prolonged progressive dysphagia, voice problems, odynophagia, and weight loss. These conditions are commonly seen in patients with esophageal cancer although barium and endoscopic examination for diagnostic purposes (Thrumurthy et al., 2019) have not been done due to financial constraints. Medical history indicates a provider at a local facility had recommended and referred the patient to Cancer treatment center in Dar Es Salaam for chemotherapy and radiotherapy. There appeared to be a gap in a provider’s recommendation as neither the financial capacity of the family was assessed, nor palliative care recommended. Consequently, the family could not manage the cost of travel, lodging and care at the urban-based Cancer treatment center located more than one-thousand kilometers away. This patient came to our attention as a family member visited us seeking for a provider who can conduct malaria and urine test at home because he had developed high grade fever. During the initial home visit, palliative care was deemed crucial, and it became the main hope in an attempt to relieve symptoms and improved the quality of patients’ life. A team from our PHC facility was mobilized to support the family and offer palliative care on a daily basis. We also engaged religious leaders from a local church to offer spiritual support that was requested by the family. This is in recognition that spiritual support is one of the key components of palliative care (Moszynski, 2011; Ntizimira et al., 2014). Our inquiry for more information from the public PHC facility where the patient had been initially managed indicated that providers have inadequate knowledge and skills and are poorly equipped to offer palliative and supportive care that often-necessitates frequent home visits. We continued to offer palliative care until the patient died few months later.

These personal experiences and many others suggest that formalized palliative care is highly needed in low-income rural communities of Africa. However, palliative care appears to be a new concept and most healthcare workers, particularly in rural PHC settings are poorly equipped to offer such care.

## Discussion

Palliative care has been documented to be one of the cornerstones of improvement of quality of life among patients facing the problems associated with life-threatening illness and their families. Palliative care involves the prevention and relief of suffering through early identification and treatment of pain and other problems impacting the physical, psychosocial, and spiritual wellbeing of the patients (Nanney et al., 2010; Moszynski, 2011; Ntizimira et al., 2014). One of the challenges with palliative care in Africa is that it appears to be relatively, a new concept and it is not always integrated within the existing healthcare systems (Nanney et al., 2010; Moszynski, 2011; Ntizimira et al., 2014). Consequently, health care providers are poorly equipped with the knowledge, skills, and resources to address all the palliative care needs of patients with chronic and life-threatening conditions in particular within low-income communities. As a result, patients in low-income rural communities continue to lack the physical, psychosocial, and spiritual support that they need in their final days of life.

There appear to be some attempts to implement palliative care interventions in Tanzania. Reports indicate that Tanzania is among the four countries in Africa that has integrated palliative care in healthcare systems (Nanney et al., 2010; Moszynski, 2011; Ntizimira et al., 2014). However, a review of this body of literature suggests that most of palliative care efforts in the country are limited to the people living with HIV (Nanney et al., 2010). Likewise, the implementation of free medical care for older people within public facilities as part of the Tanzania aging policy appears to be largely unsuccessful (Musa, 2016). This continues to put patients with non-HIV related chronic conditions that requires ongoing palliative care at a disadvantage.

Older people with chronic diseases in Tanzania who need palliative care appear to be neglected. The two cases presented indicate that many people of old age with chronic diseases in rural areas continue to die in sufferings that could be alleviated through provision of palliative care services. Poverty and rurality of the family exacerbate the elderly patients’ outcomes as it makes almost impossible for them to meet the cost of basic medical care. Furthermore, poverty and rurality hinder the possibility of seeking advanced care from a distant urban- based cancer treatment center in the country located over one- thousand kilometers from where the cases were reported. Similar concerns can be seen in palliative care strategies focusing on HIV/AIDS care (World Health Organization, 2002; Moszynski, 2011). Institutionalized community or home-based palliative care could offer hope and improve the quality of life among patients with chronic and painful conditions. However, neither the communities nor the healthcare providers are well equipped to offer the highly demanded services in rural settings. Until policy makers and governments fully integrate palliative care within PHC, its provision in rural settings will continue to rely on the actions of a few medical providers with self-drive, knowledge and dedication amidst the continued absence of formal institutionalized strategies for palliative care.

On top of the need for palliative care, the experiences shared point to limited efforts for primary preventive care of chronic diseases in rural communities. Literature indicates that interventions that focus on disease prevention and promotion of early use of primary care are more likely to reduce deaths, rates of illness, and costs associated with chronic illness (World Health Organization, 2002; Fleming, 2010; Kreiner and Hunt, 2014; Levine et al., 2019). Chronic diseases have negative impacts on the quality of patients’ life and their families, reducing the enjoyment of life, productive capacity, family relationships, and ballooning healthcare expenditures (Levine et al., 2019). These limitations could have long term impact on mental wellbeing of both the sick individual and families such as reducing one’s ability to meet the cost of care, cope with pain consequently aggravating the clinical progression of the disease (Kreiner and Hunt, 2014; Levine et al., 2019). When this is coupled with prevailing poverty and rurality which limits access to diagnostic and curative care, palliative care remains the only hope. Therefore, efforts for strengthening palliative care in rural communities need to include activities that promote primary prevention and access to diagnostic and curative care as well.

### Limitations

This paper is not without limitations. It is structured around personal accounts and analysis of the encounters with patients needing palliative care. More research may be needed to first establish the burden of chronic diseases in rural communities and then explore its socioeconomic impacts on low-income families. This could offer more evidence for institutionalization and fully integration of palliative care within PHC. The paper only examined encounters with two patients with no confirmatory diagnostic investigations done. It may be that patients referred here were also suffering or died from other serious co-morbidities that went undiagnosed. Finally, this paper did not tap onto encounters with younger patients who need palliative care in this rural community because a focus was only limited to encounters with older patients. This does not indicate that such patients do not exist in these settings because a handful of patients of younger age continues to receive informal palliative from our team. It is also important to note that our PHC continues to identify many other cases similar to those presented here.

### Conclusion

Palliative care continues to gain momentum in western countries (World Health Organization, 2002; Moszynski, 2011; Somma et al., 2013; Thrumurthy et al., 2019). Although the health policy in some low-income countries recognizes the need for palliative care, the practical implementation, particularly in PHC facilities in rural communities appears to be lacking. Rurality and poverty on the demand side, and knowledge, skills, and resources on the provider side, continue to hinder practical implication of palliative care efforts in the rural. A call for action is therefore made to policy makers and PHC stakeholders to develop and implement interventions for strengthening palliative care within primary health care in rural communities.
